# Vagal Afferent Stimulation Attenuates LPS-Induced Acute Lung Inflammation in Association with Reduced Spleen Weight and Increased CellTrace-Positive Signals in the Lung

**DOI:** 10.3390/life16091422

**Published:** 2026-08-27

**Authors:** Yuta Masuda, Sajjad Hossain, Marina Khatun, Risa Shimane, Nanaka Tameike, Yukari Takeda, Chikara Abe

**Affiliations:** 1 Department of Physiology, Faculty of Medical Sciences, University of Fukui, Fukui 910-1193, Japan; masuda19@u-fukui.ac.jp (Y.M.); saj-10@g.u-fukui.ac.jp (S.H.); mari2025@g.u-fukui.ac.jp (M.K.); st24047@g.u-fukui.ac.jp (R.S.); st24061@g.u-fukui.ac.jp (N.T.); yukari77@u-fukui.ac.jp (Y.T.); 2 Department of Biochemistry and Molecular Biology, Faculty of Life Science, Mawlana Bhashani Science and Technology University, Santosh, Tangail 1902, Bangladesh

**Keywords:** vagal afferent stimulation, acute lung inflammation, spleen, neuroimmune interaction, immune cell trafficking

## Abstract

Vagal afferent stimulation exerts potent anti-inflammatory effects through neuroimmune pathways involving the sympathetic nervous system and spleen. However, how vagal afferent stimulation affects splenic responses and CellTrace-positive signals in inflamed tissues remains unclear. In this study, we investigated the effects of vagal afferent electrical stimulation (aVNS) on splenic responses and acute lung inflammation induced by intratracheal lipopolysaccharide (LPS) in mice. As a preconditioning intervention, aVNS was applied to the right cervical vagus nerve 24 h before LPS administration. Pulmonary inflammation was evaluated by measuring tumor necrosis factor-α (TNF-α) levels in bronchoalveolar lavage fluid. Splenic responses were assessed by measuring spleen weight, and CellTrace-positive signals were evaluated following direct intrasplenic injection of CellTrace™ Far Red. aVNS significantly reduced BALF TNF-α levels (sham: 5443 ± 404 pg/mL, *n* = 5; aVNS: 2891 ± 756 pg/mL, *n* = 5) following LPS administration. In addition, aVNS reduced spleen weight 24 h after stimulation. Furthermore, the ratio of CellTrace Far Red-positive cells was significantly higher in the aVNS + LPS group than in the sham + LPS group (0.00504 ± 0.00056 vs. 0.00151 ± 0.00017, *n* = 4 per group). These findings indicate that vagal afferent stimulation reduces the BALF TNF-α response to LPS and is associated with reduced spleen weight and increased CellTrace-positive objects detected in lung sections. These observations suggest that changes in splenic and immune-cell dynamics may accompany the anti-inflammatory effects of vagal afferent stimulation.

## 1. Introduction

Acute lung inflammation is a major cause of morbidity and mortality worldwide and contributes substantially to the development of respiratory failure and acute respiratory distress syndrome [1,2]. The inflammatory response in the lung is characterized by rapid production of pro-inflammatory cytokines and chemokines, followed by recruitment and activation of immune cells within pulmonary tissues. Although these responses are essential for host defense, excessive inflammation can cause severe tissue injury and worsen clinical outcomes. Therefore, identification of endogenous mechanisms that regulate inflammatory responses has become an important research objective.

Accumulating evidence indicates that the nervous system plays a critical role in the regulation of immune function and inflammation [3,4]. In particular, vagal pathways have emerged as important components of neuroimmune communication [5]. Previous studies demonstrated that vagal afferent electrical stimulation (aVNS) exerts potent anti-inflammatory and organ-protective effects [6]. We showed that aVNS attenuates acute kidney injury through activation of a neural circuit involving the nucleus tractus solitarius, medullary C1 neurons, and the sympathetic nervous system [7,8]. This afferent-initiated pathway is distinct from the classical vagal efferent cholinergic anti-inflammatory pathway and primarily involves downstream sympathetic and catecholaminergic signaling [7,8]. Furthermore, activation of this pathway induces long-lasting protective effects that remain evident even when inflammatory insults are applied 24 h after neural stimulation, suggesting the existence of sustained immunomodulatory mechanisms.

Noradrenaline released from sympathetic nerve terminals and/or adrenaline secreted from the adrenal medulla activates splenic immune cells, leading to suppression of excessive inflammatory responses [3,4,5,7]. Consistent with this mechanism, disruption of sympathetic signaling abolishes the protective effects by aVNS, highlighting the importance of catecholamine-dependent modulation of splenic immunity [9]. These findings suggest that the spleen serves as a critical downstream effector organ linking neural activity to anti-inflammatory responses.

Despite the established importance of splenic immune cells in neuroimmune protection, the mechanisms by which these cells contribute to anti-inflammatory effects remain incompletely understood. In particular, it is unclear whether activation of vagal afferent pathways alters splenic immune cell dynamics and promotes the mobilization of spleen-derived immune cells to sites of inflammation. For splenic immune cells to exert protective effects in peripheral organs, they would be expected to leave the spleen and efficiently migrate to inflamed tissues. However, direct evidence supporting such a process is currently lacking.

Therefore, in the present study, we investigated the relationship between aVNS and splenic responses in a mouse model of LPS-induced acute lung inflammation. We first examined whether aVNS alters spleen size, which may reflect changes in splenic immune cell dynamics. We then administered a fluorescent tracer directly into the spleen to examine whether aVNS alters CellTrace-positive signals detected in inflamed lung tissue. We hypothesized that aVNS reduces spleen weight and alters CellTrace-positive signals detected in the lung following intrasplenic tracer administration.

## 2. Materials and Methods

### 2.1. Animals

All animal procedures were conducted in accordance with the Guiding Principles for the Care and Use of Animals in the Field of Physiological Science established by the Physiological Society of Japan. All experimental protocols were approved by the Animal Research Committee of the University of Fukui (Approval No. R08014). Male C57BL/6J mice (8 weeks old) were purchased from Sankyo Labo Service Corporation. A total of 31 mice were used in this study. Male mice were used to minimize sex-related variability, including potential influences of sex hormones on cardiovascular and immune responses. Animals were housed under controlled temperature (22–24 °C) and humidity conditions under a 12-h light/dark cycle and had free access to standard chow and water throughout the study unless otherwise specified.

### 2.2. Anesthesia and Postoperative Management

**Figure 1:**
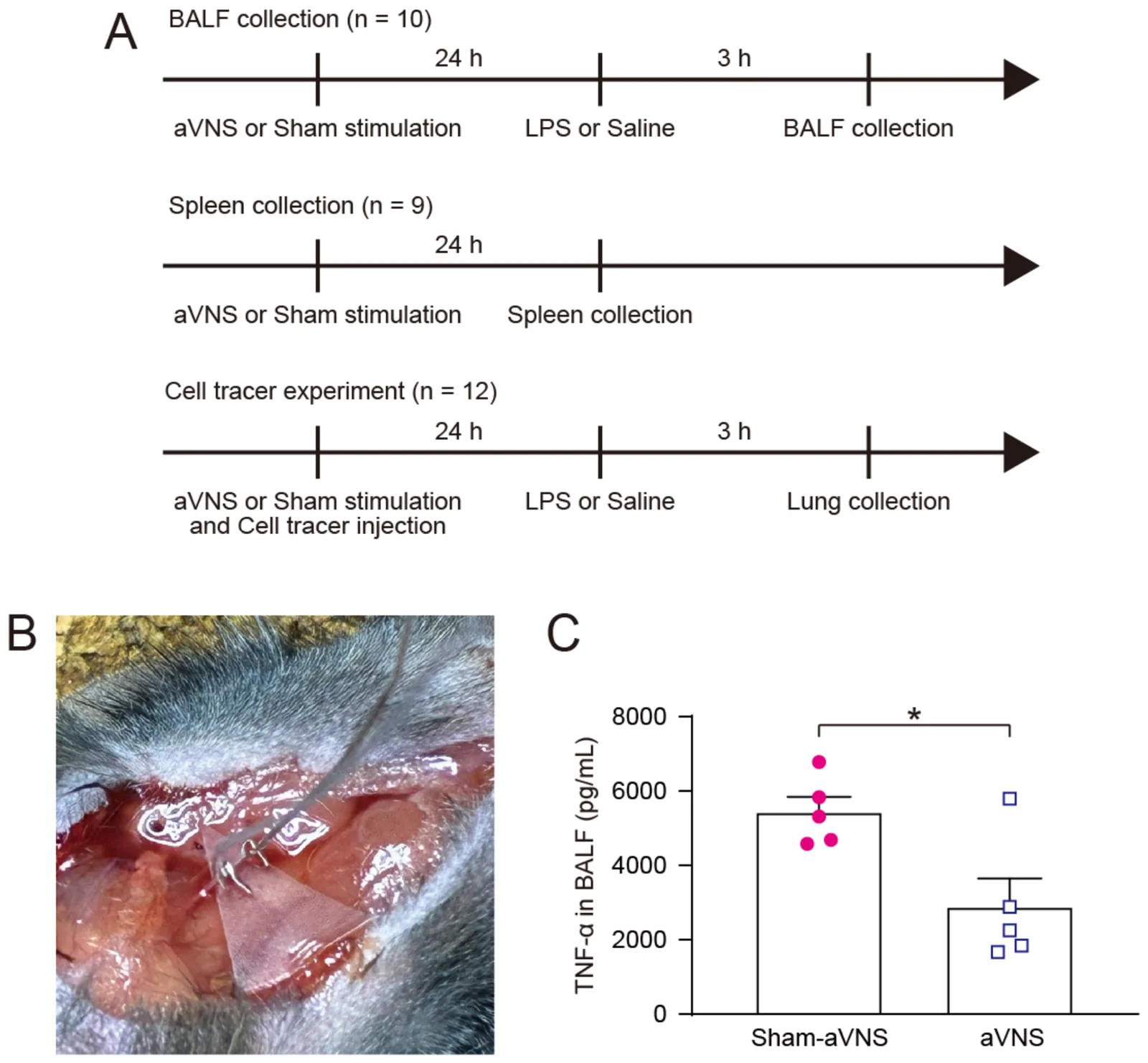
Vagal afferent stimulation attenuates LPS-induced acute lung inflammation. (**A**) Experimental timelines. (**B**) Representative photograph of the experimental setup used for vagal afferent electrical stimulation (aVNS). The right cervical vagus nerve was isolated, anesthetized distal to the stimulation site, and electrically stimulated through a bipolar electrode to selectively activate vagal afferent fibers. (**C**) Tumor necrosis factor-α (TNF-α) concentrations in bronchoalveolar lavage fluid (BALF) collected 3 h after intratracheal administration of lipopolysaccharide (LPS) (*n* = 5 in each group). Mice received sham treatment or aVNS 24 h before LPS administration. Data are presented as mean ± SEM. Statistics: unpaired *t* test [t(8) = 2.976, *p* = 0.0177]. * *p* < 0.05.

All surgical procedures were performed under aseptic conditions. For aVNS, bronchoalveolar lavage fluid (BALF) collection, and intrasplenic tracer injection, mice were anesthetized with a mixture of ketamine (120 mg/kg, i.p.) and xylazine (12 mg/kg, i.p.) (Figure 1A). Adequate anesthetic depth was confirmed by the absence of corneal and hind-paw withdrawal reflexes. Supplemental doses (10% of the initial dose, i.p.) were administered when necessary. Body temperature was maintained at 37.0 ± 0.5 °C using a servo-controlled heating pad throughout the procedures. Immediately after completion of the surgical procedure, mice received subcutaneous injections of atipamezole (2 mg/kg), penicillin G potassium (3000 U/kg), and ketoprofen (4 mg/kg). Mice were monitored during recovery from anesthesia and were returned to their home cages only after recovery of spontaneous movement and normal posture. Animals were subsequently monitored for signs of pain or distress.

For intratracheal administration of LPS, anesthesia was induced and maintained with isoflurane (2–3% in oxygen). Following completion of the procedure, mice were returned to their home cages and monitored until full recovery. Three hours after LPS administration, mice were deeply anesthetized with ketamine (240 mg/kg, i.p.) and xylazine (24 mg/kg, i.p.), corresponding to twice the doses used for surgical anesthesia. Adequate depth of anesthesia was confirmed by the absence of corneal and hind-paw withdrawal reflexes. BALF and tissue samples were then collected as described below. Death was confirmed by cessation of spontaneous respiration, followed by cervical dislocation as a secondary physical method.

All procedures were designed to minimize animal pain and distress and were performed in accordance with the principles of Replacement, Reduction, and Refinement (3Rs). Because the present study examined integrated interactions among the vagal afferent pathway, spleen, and lung, these systemic neuroimmune responses could not be adequately reproduced using an in vitro alternative. Spleen weight was used as a simple quantitative measure of the splenic response, whereas lung tissue collection was required to evaluate CellTrace-positive objects. The number of animals was limited to that required to address the experimental objectives, and multiple samples and outcome measures were obtained from individual animals whenever possible to reduce overall animal use.

### 2.3. Vagal Afferent Electrical Stimulation

aVNS was performed as previously described [6]. Briefly, mice were anesthetized with ketamine (120 mg/kg, i.p.) and xylazine (12 mg/kg, i.p.), and the right cervical vagus nerve was exposed through a midline neck incision. To selectively stimulate vagal afferent fibers, lidocaine was applied to the vagus nerve distal to the stimulation site to block efferent nerve conduction. A bipolar stainless-steel electrode (AS633; Cooner Wire, Chatsworth, CA, USA) was placed on the vagus nerve proximal to the lidocaine-treated site, and electrical stimulation was delivered using an isolated pulse stimulator.

Electrical stimulation was applied at 50 μA, 1 Hz, with a pulse duration of 1 ms for 10 min as previously established [6]. Sham-operated mice underwent identical vagus nerve isolation and distal lidocaine application but did not receive electrical stimulation. Following stimulation, the incision was closed, and the animals were allowed to recover. Twenty-four hours after aVNS, body weight and spleen weight were measured, or acute lung inflammation was induced by intratracheal administration of LPS, depending on the experimental protocol.

### 2.4. Induction of Acute Lung Inflammation

Acute lung inflammation was induced by intratracheal administration of LPS, as previously described [9,10]. Briefly, a tracheal tube constructed from polyethylene tubing (PE-10 and PE-50; Becton, Dickinson and Company, Franklin Lakes, NJ, USA) was inserted through the oral cavity into the trachea. LPS (*Escherichia coli* O111; Sigma-Aldrich, St. Louis, MO, USA) was dissolved in sterile saline at a concentration of 0.1 mg/mL and administered intratracheally at a volume of 100 μL. Control animals received an equivalent volume of sterile saline. Three hours after LPS administration, mice were euthanized for collection of BALF, spleen, and lung tissues.

### 2.5. Bronchoalveolar Lavage Fluid Collection and TNF-α Measurement

BALF was collected 3 h after intratracheal administration of LPS. Following tracheotomy, the lungs were gently lavaged with 800 μL of sterile saline through a tracheal tube. BALF samples were centrifuged, and the supernatants were collected and stored at −80 °C until analysis. The concentration of tumor necrosis factor-α (TNF-α) in BALF was measured using a commercially available enzyme-linked immunosorbent assay (ELISA) kit (Thermo Fisher Scientific, Waltham, MA, USA) according to the manufacturer’s instructions.

### 2.6. Detection of Celltrace-Positive Objects in Lung Tissue

To evaluate CellTrace-positive signals in the lung following intrasplenic tracer administration, mice were anesthetized with ketamine (120 mg/kg, i.p.) and xylazine (12 mg/kg, i.p.). The spleen was exposed through a left flank incision, and 1 μL of CellTrace™ Far Red Cell Proliferation Kit reagent (Thermo Fisher Scientific, Waltham, MA, USA) prepared as a 10 mM stock solution in DMSO and diluted to 1 mM in sterile saline immediately before use (final DMSO concentration, 10%) was injected directly into the splenic parenchyma using a microsyringe. Immediately after tracer injection, vagal afferent electrical stimulation (aVNS) was performed as described above. Sham-operated mice underwent identical procedures without electrical stimulation.

Twenty-four hours later, acute lung inflammation was induced by intratracheal administration of lipopolysaccharide (LPS). Three hours after LPS administration, mice were transcardially perfused with 100 mL phosphate-buffered saline (PBS), followed by 50 mL of 4% paraformaldehyde. Lungs were harvested, post-fixed overnight, cryoprotected in 20% sucrose, embedded in OCT compound, and sectioned at 10 μm using a cryostat. Sections were mounted with DAPI-containing mounting medium and imaged using a fluorescence microscope (BZ-X800, KEYENCE, Osaka, Japan).

For quantitative analysis, nine randomly selected fields were analyzed from each lung section using BZ-X800 Analyzer software (BZ-X800 Analyzer, KEYENCE, Osaka, Japan). Lung parenchymal area was determined from DAPI fluorescence images using a fixed threshold. Blood vessels were manually excluded from the analysis, and the remaining DAPI-positive area was defined as the DAPI-derived area. CellTrace™ Far Red-positive objects detected within the analyzed lung region were manually counted. Objects visually identified within apparent blood vessels were excluded from the analysis. The number of tracer-positive objects was normalized to the DAPI-derived area (Far Red-positive objects/μm^2^), and the mean value from the nine fields was used for statistical analysis.

### 2.7. Statistical Analysis

Statistical analyses were performed using GraphPad Prism version 11 (GraphPad Software, San Diego, CA, USA). Normality was assessed using the Shapiro–Wilk normality test. Comparisons between two groups were performed using an unpaired *t*-test. Multiple-group comparisons were analyzed using one-way analysis of variance (ANOVA) followed by Tukey’s multiple-comparison test. Data are presented as mean ± standard error of the mean (SEM). Statistical significance was defined as *p* < 0.05.

## 3. Results

### 3.1. Avns Attenuated Lps-Induced Acute Lung Inflammation

Figure 1B shows the experimental setup used for aVNS. To determine whether aVNS exerts anti-inflammatory effects in the lung, acute lung inflammation was induced by intratracheal administration of LPS 24 h after aVNS. As shown in Figure 1C, the concentration of TNF-α in BALF was significantly lower in the aVNS group than in the sham group.

### 3.2. Avns Reduces Spleen Weight 24 h After Stimulation

**Figure 2:**
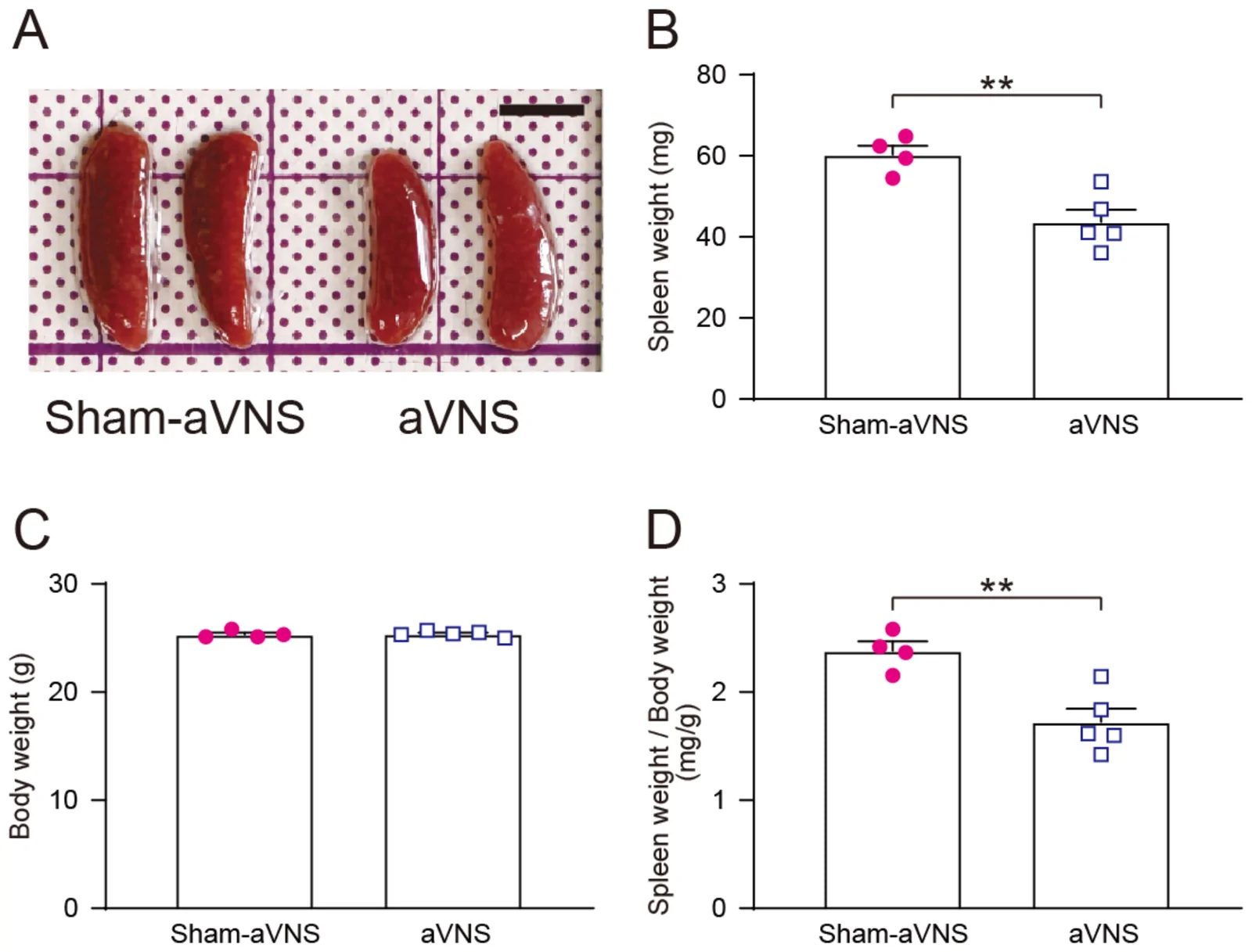
Vagal afferent stimulation reduces spleen weight. (**A**) Representative images of spleens collected 24 h after sham treatment or vagal afferent electrical stimulation (aVNS). Scale bar = 5 mm. (**B**–**D**) Spleen weight (**B**), body weight (**C**), and spleen weight normalized to body weight (**D**) measured 24 h after sham treatment or aVNS (*n* = 4 in Sham-aVNS and *n* = 5 in aVNS). Data are presented as mean ± SEM. Statistics: unpaired *t* test [t(7) = 4.228, *p* = 0.0039] in (**B**) and [t(7) = 4.092, *p* = 0.0046] in (**D**). ** *p* < 0.01.

To investigate whether aVNS alters splenic responses, spleens were collected 24 h after stimulation. Representative images of spleens obtained from sham- and aVNS-treated mice are shown in Figure 2A. Spleens from aVNS-treated mice appeared visibly smaller than those from sham-treated mice. Quantitative analysis confirmed that spleen weight was significantly lower in the aVNS group than in the sham group (Figure 2B). In contrast, body weight did not differ between the two groups (Figure 2C). Consequently, spleen weight normalized to body weight was also significantly reduced following aVNS (Figure 2D).

### 3.3. Avns Increases Celltrace-Positive Objects Detected in Lung Sections

**Figure 3:**
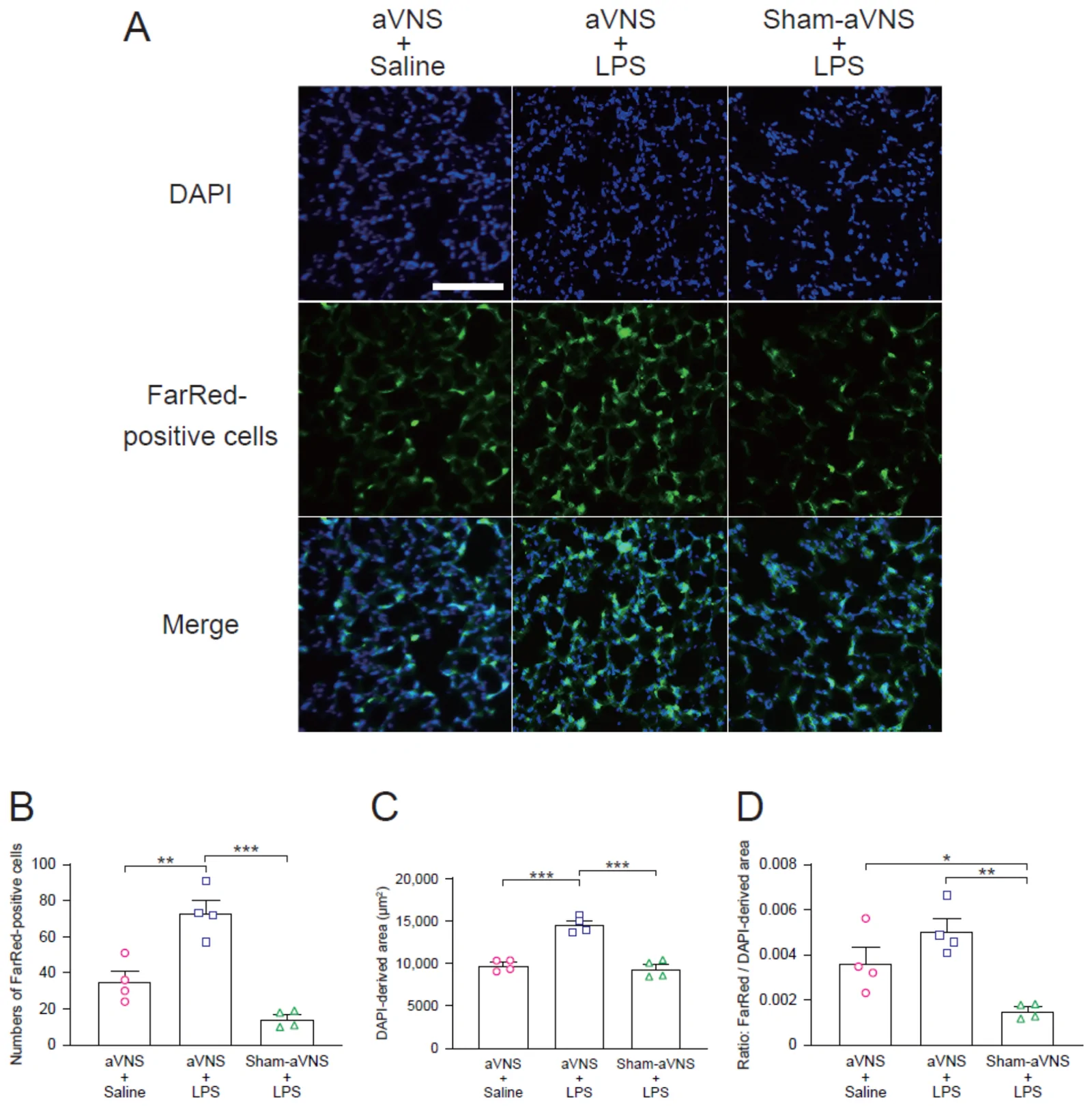
Vagal afferent stimulation increases CellTrace-positive signals detected in lung sections. (**A**) Representative fluorescence images of lung sections showing CellTrace™ Far Red-positive objects (green) and DAPI-stained nuclei (blue). Scale bar = 100 μm. (**B**–**D**) Quantitative analysis of the number of CellTrace™ Far Red-positive cells in lung sections (**B**), quantitative analysis of the DAPI-derived area determined from DAPI fluorescence images (**C**), and ratio of CellTrace™ Far Red-positive cells (pseudo-colored in green) to DAPI-derived area (**D**) (*n* = 4 in each group). Cell numbers were normalized to the DAPI-derived area to account for LPS-induced changes in lung morphology. Data are presented as mean ± SEM. Statistics: one-way ANOVA followed by Tukey’s multiple-comparison test. [F (2, 9) = 30.48; *p* < 0.0001] in (**B**), [F (2, 9) = 41.95; *p* < 0.0001] in (**C**), and [F (2, 9) = 11.41; *p* = 0.0034] in (**D**). * *p* < 0.05, ** *p* < 0.01, *** *p* < 0.001.

To examine whether aVNS affects CellTrace Far Red-positive signals in lung tissue, CellTrace™ Far Red was injected directly into the spleen before electrical stimulation. Representative fluorescence images of lung sections are shown in Figure 3A. Only a small number of Far Red-positive cells were observed in the lungs of mice treated with aVNS alone. In contrast, the number of Far Red-positive cells markedly increased following LPS administration in mice that received aVNS, whereas substantially fewer labeled cells were detected in the lungs of sham-treated mice after LPS challenge.

Quantitative analysis confirmed that the number of Far Red-positive cells was significantly greater in the aVNS + LPS group than in both the aVNS + saline and sham-aVNS + LPS groups (Figure 3B). The DAPI-derived area was significantly greater in the aVNS + LPS group than in the other groups (Figure 3C). Therefore, the number of Far Red-positive cells was normalized to the DAPI-derived area. After normalization, the ratio of Far Red-positive cells to DAPI-derived area was significantly higher in the aVNS + LPS group than in the sham-aVNS + LPS group, but did not differ significantly from the aVNS + saline group (Figure 3D).

## 4. Discussion

The present study demonstrated that (1) aVNS reduces the LPS-induced increase in BALF TNF-α, (2) aVNS reduces spleen weight 24 h after stimulation, and (3) aVNS is associated with changes in the number of CellTrace-positive objects detected in lung tissue.

The finding that aVNS reduced TNF-α levels in bronchoalveolar lavage fluid is consistent with previous studies demonstrating that vagal stimulation attenuates acute lung inflammation [11,12,13]. Our group previously reported that aVNS protects against acute kidney injury through activation of a neural circuit involving medullary C1 neurons and the sympathetic nervous system [6,7], and that direct activation of C1 neurons suppresses LPS-induced pulmonary inflammation [9]. Together with these findings, the present results support the concept that activation of the aVNS–sympathetic pathway induces a sustained anti-inflammatory state that remains effective even when inflammatory insults occur 24 h after neural stimulation. Importantly, the present study further showed that aVNS was associated with reduced spleen weight and altered CellTrace-positive signals in lung tissue. These observations might provide new insight into the cellular mechanisms linking neural stimulation to peripheral anti-inflammatory protection.

The spleen is increasingly recognized as a key organ in neuroimmune communication and is required for the protective effects of autonomic neural stimulation in several inflammatory conditions. Previous studies from our group and others have demonstrated that sympathetic activation and catecholamine release are essential mediators of neuroimmune protection [6,7,9,14,15]. Consistent with these observations, aVNS reduced spleen weight 24 h after stimulation. Given the sympathetic innervation of the spleen, one possible explanation is sympathetically mediated splenic contraction, which could alter splenic blood volume and potentially influence splenic cell dynamics. However, because splenic histology, total splenocyte number, and immune-cell composition were not examined, the present findings cannot distinguish splenic contraction from changes in cellular content or other mechanisms.

The spleen is innervated predominantly by sympathetic nerves and lacks direct parasympathetic (vagal efferent) innervation [7,16,17]. Therefore, the reduction in spleen weight observed after aVNS is likely mediated through activation of the sympathetic nervous system rather than direct vagal signaling and may reflect mobilization of catecholamine-responsive immune cells from the spleen. Indeed, previous studies have indicated that sympathetic and adrenergic signaling influence immune cell trafficking during inflammatory responses [18]. Moreover, previous studies have demonstrated that splenic monocytes can be mobilized to injured peripheral tissues, including the myocardium and lung [19,20]. On the other hand, we previously demonstrated that adoptive transfer of splenic CD4-positive T cells pretreated with the β2-adrenergic receptor agonist salbutamol attenuated acute lung inflammation in recipient mice [9]. Together with previous reports demonstrating that adrenergic signaling regulates immune cell activation [21,22,23], these findings suggest that catecholamine signaling alters the functional properties of splenic immune cells and enables them to exert anti-inflammatory effects in distant organs. Although the origin, phenotype, and function of the CellTrace-positive objects detected in the lung remain unknown, the present observations raise the possibility that alterations in splenic and immune-cell dynamics accompany the anti-inflammatory effects of aVNS.

Several limitations of this study should be acknowledged. First, the identity of the CellTrace-positive objects detected in lung sections was not determined. Moreover, free CellTrace dye may have entered the circulation and labeled circulating, resident, or infiltrating cells independently of spleen-to-lung cell trafficking; therefore, the origin of the CellTrace-positive objects cannot be definitively established. Future studies using flow cytometry or immunohistochemical analyses will be necessary to identify the immune cell populations involved. Second, although changes in CellTrace-positive objects were observed together with reduced BALF TNF-α levels, a causal relationship was not directly examined. In addition, the reduction in spleen weight cannot be interpreted as evidence of structural remodeling or cell mobilization because splenic histology, total splenocyte counts, and immune-cell composition were not assessed. Furthermore, because a sham + saline group was not included, the independent effects of aVNS and LPS and their interaction on CellTrace-positive signals could not be determined. Third, although the DAPI-derived area differed among experimental groups, this parameter was measured primarily to normalize the number of tracer-labeled cells and was not intended as a quantitative assessment of lung injury or tissue repair. Because DAPI staining was used as a surrogate to delineate lung tissue structure, changes in cellularity associated with inflammation may have influenced this measurement. Therefore, whether the increased DAPI-derived area observed in the aVNS-treated mice reflects preservation or early restoration of alveolar architecture requires further histopathological investigation. Finally, the molecular mechanisms underlying the reduced spleen weight and altered CellTrace-positive signals following aVNS remain to be elucidated.

In conclusion, vagal afferent stimulation was associated with a reduced BALF TNF-α response to LPS, reduced spleen weight, and altered CellTrace-positive signals in lung tissue. These findings suggest that changes in splenic and immune-cell dynamics may accompany the anti-inflammatory effects of vagal afferent stimulation; however, their causal and functional relationships remain to be established.

## Data Availability

The data presented in this study are available on request from the corresponding author.
